# Cannabigerol Prevents Quorum Sensing and Biofilm Formation of *Vibrio harveyi*

**DOI:** 10.3389/fmicb.2020.00858

**Published:** 2020-05-07

**Authors:** Muna Aqawi, Ruth Gallily, Ronit Vogt Sionov, Batya Zaks, Michael Friedman, Doron Steinberg

**Affiliations:** ^1^The Biofilm Research Laboratory, The Faculty of Dental Medicine, The Institute of Dental Sciences, The Hebrew University of Jerusalem, Jerusalem, Israel; ^2^The Lautenberg Center for General and Tumor Immunology, The Hadassah Medical School, The Hebrew University of Jerusalem, Jerusalem, Israel; ^3^The Department of Pharmaceutics, The Faculty of Medicine, The Institute of Drug Research, The Hebrew University of Jerusalem, Jerusalem, Israel

**Keywords:** biofilm, bioluminescence, cannabinoids, motility, quorum sensing, *Vibrio harveyi*

## Abstract

Cannabigerol (CBG) is a non-psychoactive cannabinoid naturally present in trace amounts in the Cannabis plant. So far, CBG has been shown to exert diverse activities in eukaryotes. However, much less is known about its effects on prokaryotes. In this study, we investigated the potential role of CBG as an anti-biofilm and anti-quorum sensing agent against *Vibrio harveyi*. Quorum sensing (QS) is a cell-to-cell communication system among bacteria that involves small signaling molecules called autoinducers, enabling bacteria to sense the surrounding environment. The autoinducers cause alterations in gene expression and induce bioluminescence, pigment production, motility and biofilm formation. The effect of CBG was tested on *V. harveyi* grown under planktonic and biofilm conditions. CBG reduced the QS-regulated bioluminescence and biofilm formation of *V. harveyi* at concentrations not affecting the planktonic bacterial growth. CBG also reduced the motility of *V. harveyi* in a dose-dependent manner. We further observed that CBG increased *LuxO* expression and activity, with a concomitant 80% downregulation of the *LuxR* gene. Exogenous addition of autoinducers could not overcome the QS-inhibitory effect of CBG, suggesting that CBG interferes with the transmission of the autoinducer signals. In conclusion, our study shows that CBG is a potential anti-biofilm agent via inhibition of the QS cascade.

## Introduction

Bacteria can communicate by means of signaling molecules called autoinducers (AIs) in a process called quorum sensing (QS). Quorum sensing governs several processes which are critical for bacterial survival and allows them to respond to changes in cell density (Mukherjee and Bassler, 2019). These processes include biofilm formation, virulence factor secretion, bioluminescence, motility, antibiotic production, sporulation and development of genetic competence (Singh et al., 2009). Quorum sensing allows bacteria to switch between two distinct gene expression programs, one that is favored at low cell density (LCD) for individual, asocial behaviors, while the other is favored at high cell density (HCD) for social, group behaviors (Ng and Bassler, 2009).

The free living, marine quorum-sensing bacterium *Vibrio harveyi* produces at least three AIs, harveyi autoinducer 1 (HAI-1; acylated homoserine lactone; AHL), autoinducer 2 (AI-2; furanosyl borate diester), and cholera autoinducer 1 (CAI-1; a long-chain amino ketone (Z)-3-aminoundec-2-en-4-one) that interact with their respective receptors LuxN, LuxP/Q and CqsS (Chen et al., 2002; Zhang et al., 2012; Soni et al., 2015). The autoinducers elicit signal transduction pathways in *V. harveyi* converging in the expression of bioluminescence and biofilm formation (Waters and Bassler, 2006). When no or low quantities of autoinducers are present (at low cell density), the receptors autophosphorylate and transfer phosphate to LuxO through LuxU. Phosphorylated LuxO in combination with the sigma factor σ^54^ activates the transcription of the genes encoding five regulatory small RNAs (qrr1-5) (Tu and Bassler, 2007). The qrr sRNAs together with the RNA-binding protein Hfq inhibit the translation of the mRNA of the master QS regulator LuxR. LuxO also reduces LuxR activity by inducing the expression of AphA (Rutherford et al., 2011). Therefore, at LCD, LuxR protein is not produced and there is no bioluminescence. In contrast, when the autoinducer concentrations are high (at high cell density), the receptors switch to phosphatases allowing for the dephosphorylation of LuxU and LuxO. This in turn results in LuxR-mediated induction of genes involved in bioluminescence and biofilm formation (Ng and Bassler, 2009; Zhang et al., 2012).

**FIGURE 1:**
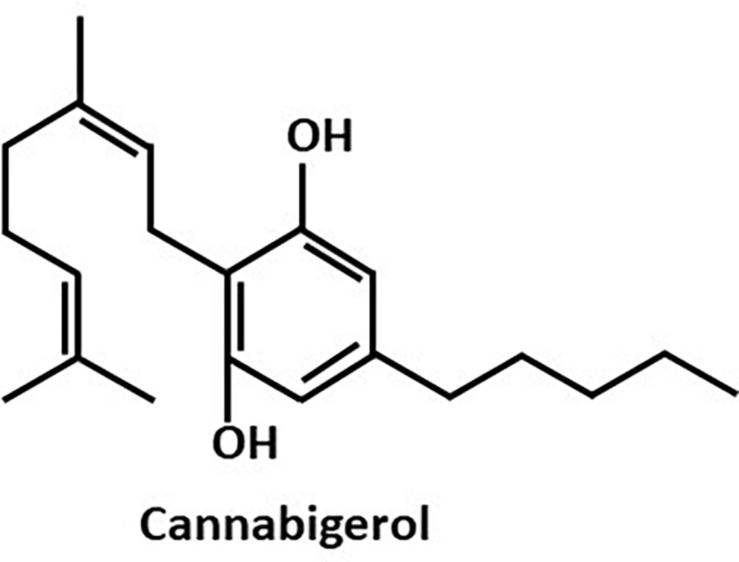
The chemical structure of Cannabigerol (CBG).

Since anti-QS compounds are known to have the ability to prohibit bacterial pathogenicity, research is currently directed toward disrupting QS as an attractive target for the development of novel anti-infective agents that do not rely on the use of antibiotics (Asfour, 2018). We have previously shown interference of the bacterial signal-transduction system by the synthetic cannabinoid receptor agonist HU-210 (Soni et al., 2015) and the endocannabinoid anandamide (Friedman et al., 2019). Here, we studied the anti-biofilm and anti-QS effects of the *Cannabis sativa* plant component cannabigerol (CBG) (Figure 1) using the marine biofilm-producing bacterial species *V. harveyi*. The phytocannabinoids of *C. sativa* have been shown to exert potential therapeutic activities in mammalians (Turner et al., 2017). Specifically, CBG exerts anti-inflammatory, neuroprotective and anti-tumor properties (Eisohly et al., 1982), and has been shown to be effective in the treatment of glaucoma, psoriasis, dry-skin syndrome and pain (Olah et al., 2016). In addition, it elicited hyperphagia (Brierley et al., 2016) and attenuated colitis (Borrelli et al., 2013) in mice. On the contrary, much less is known about its effects on prokaryotes. A previous study showed that CBG displays antibacterial properties against methicillin-resistant *Staphylococcus aureus* strains (Appendino et al., 2008). Here we tested the anti-quorum sensing and anti-biofilm formation potential of CBG on *V. harveyi*.

## Materials and Methods

### Materials

Cannabigerol (CBG) (hemp isolate, 98.5% purity) was purchased from NC Labs (Czech Republic) and dissolved in ethanol at a concentration of 18 mg/ml. As control, respective dilutions of ethanol corresponding to the different CBG concentrations were used. (S)-4,5-Dihydroxy-2,3-pentandione (DPD, AI-2) was purchased from OMM Scientific Inc.

### Bacterial Growth and Biofilm Formation

*V. harveyi* wild-type strain BB120 was obtained from ATCC (*Vibrio campbellii* BAA-1116^TM^). The mutant bacterial strains were generously provided by Prof. B. Bassler (Princeton University) (Table 1; Bassler et al., 1993, 1994, 1997; Freeman and Bassler, 1999; Surette et al., 1999; Mok et al., 2003). For planktonic growth, the *V. harveyi* strains were incubated aerobically in complete autoinducer bioassay (AB) medium (300 mM NaCl, 50 mM MgSO_4_, 2 mg/ml Casamino acids, 0.5 mM L-arginine, 20 μg/ml thiamine, 2 μg/ml riboflavin, 5 mM potassium phosphate and 0.5% glycerol; pH 7.5) at 30°C for 20–24 h. For biofilm formation, the bacteria were grown in complete AB medium supplemented with higher concentrations of thiamine (0.3 mg/ml) and riboflavin (0.3 μg/ml).

**TABLE 1:** *Vibrio harveyi* wild-type (wt) and mutant strains carrying genetic defects in the quorum sensing genes.

**Strain**	**Sensor1 (LuxN)**	**Sensor2 (LuxP/LuxQ)**	**AI-1**	**AI-2**	**Gene**	**References**
BB120 (wt)	+	+	+	+	Wild-type	Bassler et al., 1997
BB170	–	+	+	+	*luxN*^–^	Bassler et al., 1993
BB152	+	+	–	+	*luxM ^–^*	Bassler et al., 1994
BB960	+	–	+	+	*luxQ*^–^	Bassler et al., 1994
BB886	+	–	+	+	*luxP*^–^	Bassler et al., 1994
MM77	+	+	–	–	*luxM ^–^*, *luxS*^–^	Mok et al., 2003
MM30	+	+	+	–	*luxS*^–^	Surette et al., 1999
MM32	–	+	+	–	*luxS*^–^, *luxN ^–^*	Surette et al., 1999
JAF 375	–	–	+	+	*luxQ*^–^, *luxN ^–^*	Freeman and Bassler, 1999

### DNA Quantification by Quantitative (q) PCR

DNA extraction and quantification were performed as previously described (Periasamy and Kolenbrander, 2009; Assaf et al., 2014). Briefly, biofilm was allowed to form in the absence or presence of different concentrations of CBG and ethanol in polystyrene 48-well tissue culture plates (Nunc). At the end of incubation, the growth medium was removed, and the formed biofilms were washed twice with PBS. Then, 200 μl of NaOH (0.04 M) was added to each well and the plates were incubated in a hot water bath for 1 h at 60°C, followed by neutralization with 18.5 μl Tris:HCl pH 7.0. The amount of DNA in each sample was quantified by qPCR with specific primers for *V. harveyi* 16S rRNA (Table 2) using a Bio-Rad CFX Connect Real-time system and the Bio-Rad CFX Maestro program. The amount of DNA in each sample was calculated according to the standard curve obtained using known DNA concentrations of purified *V. harveyi* DNA. Purified DNA was extracted from an overnight culture of *V. harveyi* BB120 using GenElute Bacterial Genomic DNA kit (Sigma Aldrich, St. Louis, MO, United States) as per the manufacturer’s instructions.

**TABLE 2:** Primers used for real-time PCR.

**Gene**	**Forward primer**	**Reverse primer**
16S rRNA	GAGTTCGGTTTCTTTCAA	TGTAGTTTTTCGCTAATTTC
LuxR	TCAATTGCAAAGAGACCTCG	AGCAAACACTTCAAGAGCGA
LuxU	ATGGACTCCTACATTGGCACTT	AAGCTGGCAGCACTACTTTTC
LuxS	GGCGTACCAATCAAGCTCATGT	CGCAGGCTTTATGCGTAATCA
LuxM	ATTCTTGCCCGACTCTGGTG	CAACACTTCGCAAACGGCTT
LuxP	GTGGTTTACCCAGGACAGCA	GTTTGTCCCACTCACGGACT
LuxN	AGGTATCGGCAAAGCGTTCA	ATACGGCGATCCGCTTCAAT
LuxQ	GTCCAGCACCTGATGACGAT	TGCCCATCGCCAGTAAACTT
LuxO	TCCTAATCAAACCATGTGAAGC	GATGAAGCCTTGGTAATTTTGG
aphA	ATCCATCAACTCTAGGTGATAAACG	CGTCGCGAGTGCTAAGTACA
Hfq	CGTGAGCGTATCCCGGTATCTAT	TTGCAGTTTGATACCGTTCACAAG
qrr1	CTCGGGTCACCTATCCAACTGA	TCGGATCTATTGGCTCGTTCTG
qrr2	CTTAAGCCGAGGGTCACCTAGC	CAATTAGGGCGATTGGCTTATGT
qrr3	CTTAAGCCGAGGGTCACCTAGC	ACAAATTCGAGTCCACTAACAACGT
qrr4	CCTTATTAAGCCGAGGGTCAC	GTTGATTGGCGGTATATACTTGTG
qrr5	GACGTTGTTAGTGAACCCAATTGTT	CACAAGGTTTGTGATTGGCTGTATA

### Growth and Bioluminescence of Wild-Type and Mutant *V. harveyi* Strains

Overnight cultures (OD_595_∼0.7) of wild-type *V. harveyi* (BB120) and mutant *V. harveyi* strains were diluted 1:200 in complete AB medium, and 180 μl of the diluted bacterial cultures were transferred to each well of an optic flat-bottom white 96-well cell culture microplate (μCLEAR, Greiner Bio-One), together with 20 μl of CBG at different concentrations. The absorbance (OD_595_) and luminescence were read in parallel every 30 min for 20 h using an infinite M200PRO TECAN plate reader. The plate reader was kept at a constant temperature of 30°C throughout the experiment. The luminescence values, representing quorum sensing, were normalized by dividing them with the respective absorbance values in order to correct for differences in growth rates. The area under the curve was calculated for each sample and compared with untreated control samples as previously described (Aharoni et al., 2008; Feldman et al., 2009).

### RNA Extraction

The assay was performed similarly to the method used by Feldman et al. (2014) with slight modifications. An overnight culture of *V. harveyi* (OD_595_∼0.7) was diluted 1:10 in complete AB medium and incubated at 30°C under aerobic conditions in the absence or presence of 2 μg/ml CBG for 10 h. Eight milliliter of bacterial culture were used for each sample. At the end of incubation, the supernatant was removed, and the cells were washed with 2 ml of PBS and incubated with 2 ml of RNA Protect (Qiagen, Hilden, Germany) for 5 min at room temperature. RNA was isolated using the RNeasy MINI kit (Qiagen) including on-column DNase digestion according to the manufacturer’s instructions. RNA purity and quantity were determined using Nanodrop (Nanovue, GE Healthcare Life Sciences, Buckinghamshire, United Kingdom). Only samples with an OD_260_/OD_280_ ratio of 2 and an OD_260_/OD_230_ ratio above 2 were used for cDNA synthesis. The samples were stored at −80°C until use.

### Reverse Transcription (RT) and Quantitative Real-Time PCR

The purified RNA was reverse transcribed into cDNA using the cDNA qScript cDNA synthesis kit (QuantaBio). The relative expression levels of target genes were analyzed by Bio-Rad CFX Connect Real-time system and the Bio-Rad CFX Maestro program. Power Sybr Green PCR Master mix (Applied Biosystems) was used to amplify the genes of 10 ng cDNA per well in combination with 300 nM of respective F/R primer set (Table 2). For each set of primers, a standard amplification curve (critical threshold cycle vs. exponential of concentration) was plotted, and only those with a slope around –3.2 were used for analysis. The PCR cycle involved initial heating at 50°C for 2 min, activation step at 95°C for 10 min, followed by 40 cycles of amplification (95°C for 15 s, 60°C for 1 min), and the dissociation curve was determined by initial heating at 95°C for 15 s, followed by 10 s at 60°C, and 0.5 temperature increment until reaching 95°C. The expression of 16S rRNA was used for normalization and to calculate the relative changes in target gene expression using the 2^−ΔΔ*Ct*^ method. Gene expression was expressed in relative values, setting the expression level of the untreated control to 1 for each gene (Assaf et al., 2014; Feldman et al., 2014).

### Autoinducer Preparation

Supernatant from log-phase growing *V. harveyi* mutant strains in incomplete AB medium (300 mM NaCl, 50 mM MgSO_4_, 2 mg/ml Casamino acids, 20 μg/ml thiamine, 2 μg/ml riboflavin; pH 7.5) was collected, filtrated (0.22 μm) and kept at –20°C. The BB152 (AI-1^–^, AI-2^+^) was used for obtaining AI-2, while MM30 (AI-1^+^, AI-2^–^) was used for preparation of AI-1. Supernatant of MM77 (AI-1^–^, AI-2^–^) was used as a negative control.

### Motility of *V. harveyi*

The motility assay was performed on soft agar plates as described previously (Yang and Defoirdt, 2015). Briefly, after cooling down autoclaved AB medium with 0.2% agar to 60°C, different concentrations of CBG along with riboflavin (0.02 μg/ml) and thiamine (0.02 mg/ml) were added and poured into small petri dishes. Agar plates without CBG served as controls. Following solidification, 3 μl of overnight *V. harveyi* culture (OD_595_∼0.5) were inoculated at the center of the agar plate and incubated at 30°C for 15h. The area of the motility halo was measured using Image J software (National Institute of Health) and compared with the control.

### High Resolution Scanning Electron Microscopy (HR-SEM)

Biofilms were prepared as described above with different concentrations of CBG and corresponding ethanol dilutions. Biofilms without CBG served as additional controls. Biofilm was allowed to form on sterile circular glass pieces that were inserted into the wells of 96-well microtiter plates. After a 24 h incubation, the glass specimens were removed, rinsed with DDW and fixed in 4% glutaraldehyde for 40 min. The glass specimens were washed again with DDW and allowed to dry at room temperature. The specimens were then mounted on a metal stub and sputter coated with gold prior to SEM analysis. A high-resolution scanning electron microscope (Magellan XHR 400L, FEI Company, Holland) was used. Three specimens from each treatment group were prepared and examined under SEM to evaluate the effect of CBG on biofilm formation (Louwakul et al., 2017).

### Statistical Analysis

Three independent experiments were conducted and triplicate samples were measured within each experiment. Statistical analysis of the collected data was performed using Student’s *t*-test in Microsoft Excel. A *p* < 0.05 was considered significant.

## Results

### CBG Prevents Quorum Sensing in *V. harveyi*

**FIGURE 2:**
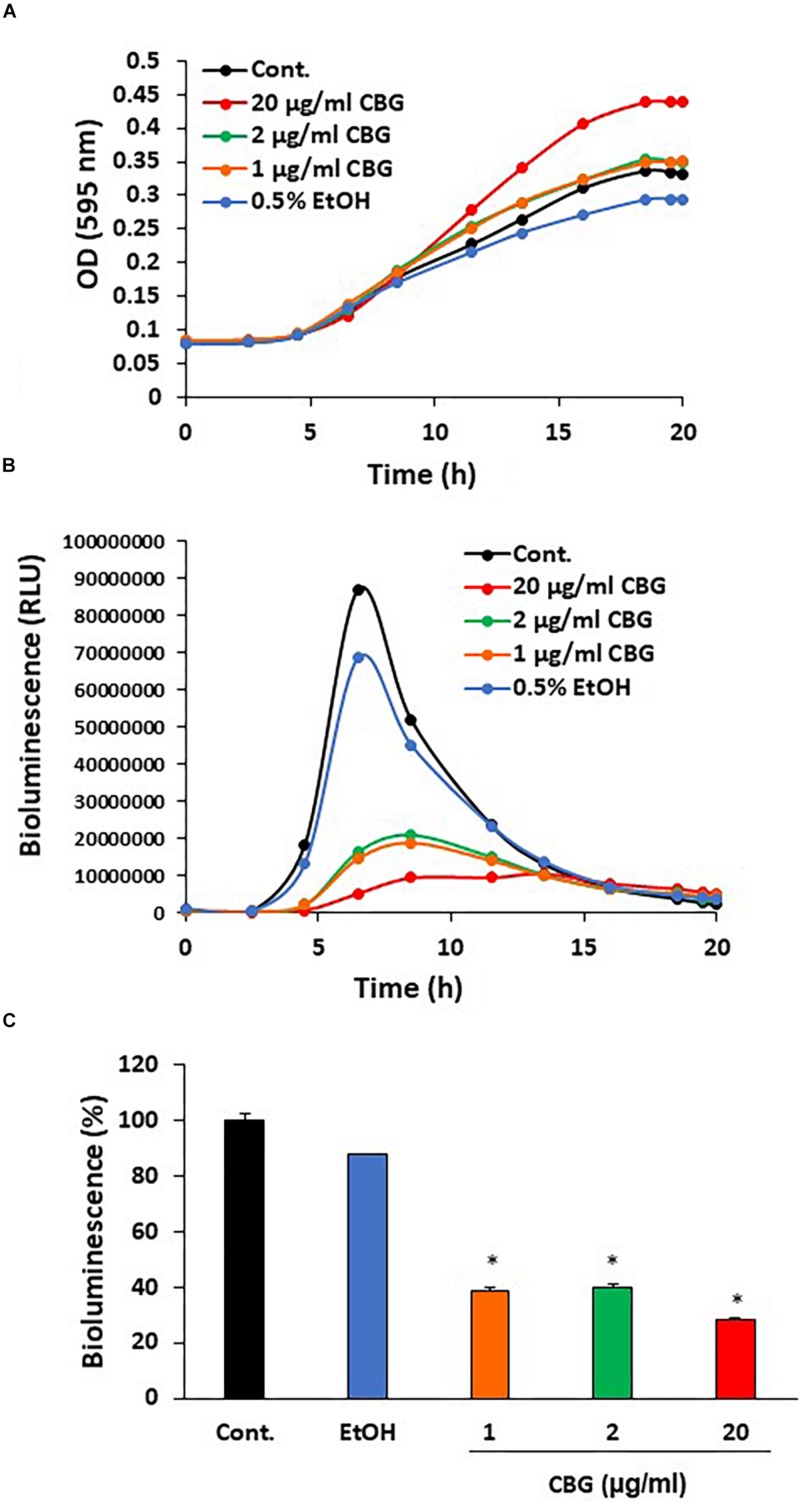
Anti-quorum sensing activity of CBG on wild-type *V. harveyi* (BB120). **(A)** CBG did not affect the planktonic growth of *V. harveyi*. **(B)** CBD strongly prevented the bioluminescence of *V. harveyi*. The graphs represent the average of 3 samples. **(C)** The relative bioluminescence as determined by the area under the curve (AUC) of the graph presented in **(B)** *N* = 3, *p < 0.05.

To explore the effect of CBG on quorum sensing of *V. harveyi* (wild-type strain BB120), the bacteria were exposed to different concentrations of CBG or the respective ethanol dilutions in complete AB growth medium and incubated for 20 h. Untreated *V. harveyi* served as an additional control. The planktonic growth was monitored by measuring the optical density (Figure 2A), and the induction of quorum sensing was monitored at the same time points by measuring bioluminescence (Figure 2B). We found that CBG at concentrations 1–20 μg/ml did not affect the bacterial growth (Figure 2A), but significantly reduced the bioluminescence (Figures 2B,C; 65 ± 4% inhibition; *p* < 0.05). Strong inhibition was already observed at 1 μg/ml CBG, with only a slightly higher inhibition when increasing the concentration to 20 μg/ml (Figures 2B,C).

### CBG Prevents Biofilm Formation by *V. harveyi*

**FIGURE 3:**
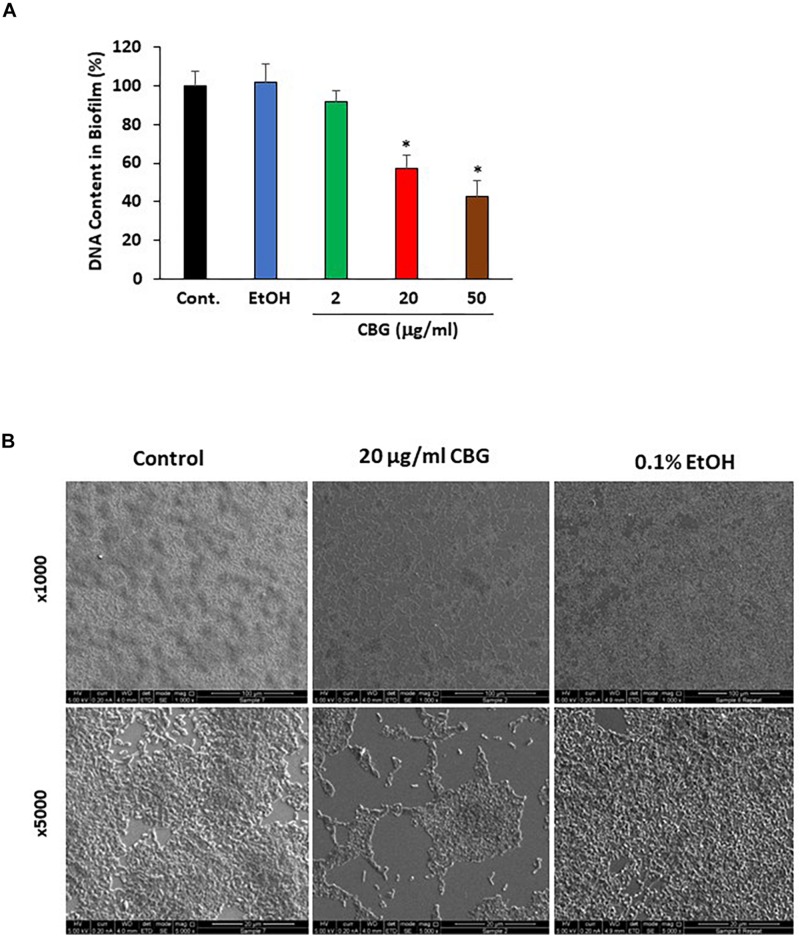
Anti-biofilm activity of CBG on wild-type *V. harveyi* (BB120). **(A)** CBG reduced the amount of DNA in the biofilms formed by *V. harveyi*. *V. harveyi* were grown in complete AB medium for 24 h in the presence of various concentrations of CBG, and the amount of DNA in the biofilms were quantified by real-time PCR using primers for 16S rRNA. *N* = 3. *p < 0.05. **(B)** Scanning electron microscopy of biofilms formed by wild-type *V. harveyi* in the presence or absence of 20 μg/ml CBG. 0.1% Ethanol (EtOH) served as vehicle control. Two different magnifications are shown (x1000 and x5000).

Bacterial biofilm formation is associated with the quorum sensing process which can be critical for bacterial survival (Rutherford and Bassler, 2012). To test the effect of CBG on wild-type *V. harveyi* biofilm formation, the DNA content in biofilms was quantified in the absence or presence of different concentrations of CBG. The percentage reduction in biofilm formation was calculated in comparison to untreated samples and samples treated with respective dilutions of ethanol. According to the collected data, CBG at 20 and 50 μg/ml reduced the amount of bacteria in the biofilms to 58 ± 7 and 43 ± 8%, respectively (Figure 3A; *p* < 0.05). To further confirm the anti-biofilm activity, biofilms of *V. harveyi* formed in the absence or presence of 20 μg/ml CBG or respective ethanol dilutions were examined under a scanning electron microscope (SEM) at a magnification of x1000–5000. As can be seen from Figure 3B, the biofilm biomass in the CBG-treated group was strongly reduced in comparison to control samples. Moreover, the structure of CBG-treated biofilm was altered. The control biofilms appeared homogeneously and firmly aggregated, while biofilm exposed to CBG appeared as dispersed small aggregates and several single cells (Figure 3B).

### CBG Reduces the Motility of *V. harveyi*

**FIGURE 4:**
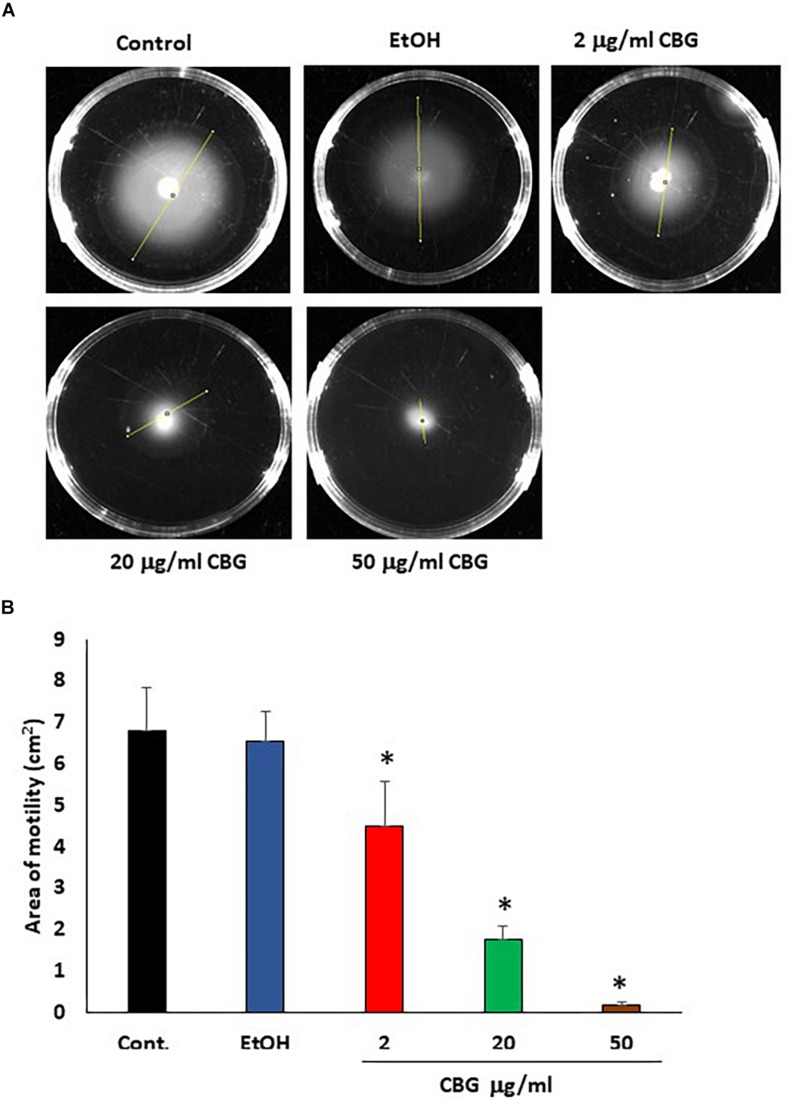
CBG inhibits *V. harveyi* motility. **(A)** The motility of *V. harveyi* in soft agar containing the indicated concentrations of CBG. **(B)** The relative motility of *V. harveyi* in the presence of indicated concentrations of CBG as determined by the spreading area shown in **(A)** *N* = 3, *p < 0.05.

Motility is another potent virulence factor which has been shown to be associated with quorum sensing (Yang and Defoirdt, 2015). Therefore, the effect of CBG on the motility of wild-type *V. harveyi* was studied. The motility halos decreased with increasing concentrations of CBG in a dose-dependent manner (Figure 4A). Quantification of motility area showed a 74 ± 5 and 97 ± 1% reduction in motility in the presence of 20 and 50 μg/ml CBG, respectively (Figure 4B; *p* < 0.05). Ethanol as high as 2% had no effect on the motility (Figure 4B).

### CBG Antagonizes the Quorum Sensing Signals Delivered by the Autoinducers

**FIGURE 5:**
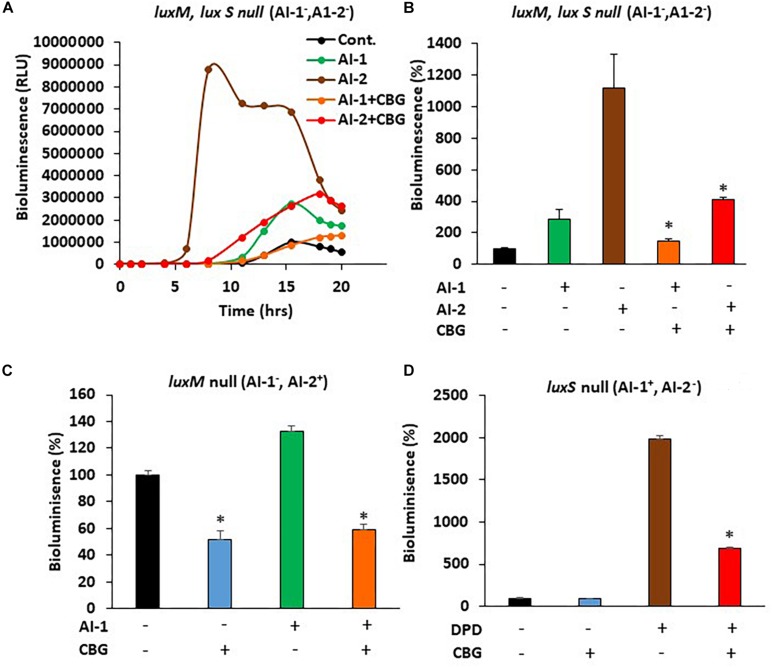
CBG antagonizes the quorum sensing signals delivered by autoinducers. **(A)** The relative bioluminescence of *luxM*, *luxS* double mutant MM77 (AI-1^–^, AI-2^–^) strain was measured over time in the absence or presence of AI-1, AI-2 and/or 1 μg/ml CBG. The bioluminescence was corrected for differences in bacterial growth by simultaneously measuring the optical density at 595 nm. **(B)** The relative bioluminescence as determined by the area under the curve (AUC) of the graph presented in **(A)** *N* = 3, *p < 0.05. **(C)** The relative bioluminescence as determined by the area under the curve (AUC) of *luxM* null mutant BB152 (AI-1^–^AI-2^+^) exposed to AI-1 and/or 1 μg/ml CBG. *N* = 3. *p < 0.05. **(D)** The relative bioluminescence as determined by the area under the curve (AUC) of *luxS* null mutant MM30 (AI-1^+^AI-2^–^) exposed to the AI-2 precursor DPD (10 μM) and/or 1 μg/ml CBG. *N* = 3, *p < 0.05.

In order to study whether CBG interferes with the quorum sensing signals delivered by the autoinducers, we exogenously added autoinducers to autoinducer-deficient mutant strains and exposed them to CBG. The bioluminescence vs. planktonic growth was monitored each 30 min for a total period of 20 h. As expected, almost no bioluminescence was emitted by the double *luxM*, *luxS* null mutant MM77 lacking both AI-1 and AI-2 (Figure 5A, black line). Exogenously added AI-2 induced a strong bioluminescence response (Figure 5A, brown line), while AI-1 caused a more moderate increase (Figure 5A, green line). This is in line with the findings of Anetzberger et al. (Anetzberger et al., 2012). Importantly, the addition of CBG led to a 47.6 ± 5.3 and 63.2 ± 1.6% reduction of the AI-1 and AI-2-induced bioluminescence, respectively (Figures 5A,B, orange and red lines/columns). Similarly, CBG inhibited the AI-1-induced bioluminescence in the *luxM* null BB152 (AI-1^–^, AI-2^+^) strain (Figure 5C) and the DPD-induced bioluminescence in the *luxS* null MM30 (AI-1^+^, AI-2^–^) strain (Figure 5D). DPD is a precursor of AI-2 that spontaneously cyclizes into AI-2 (Papenfort and Bassler, 2016).

### CBG Increases LuxO Expression and Activity

**FIGURE 6:**
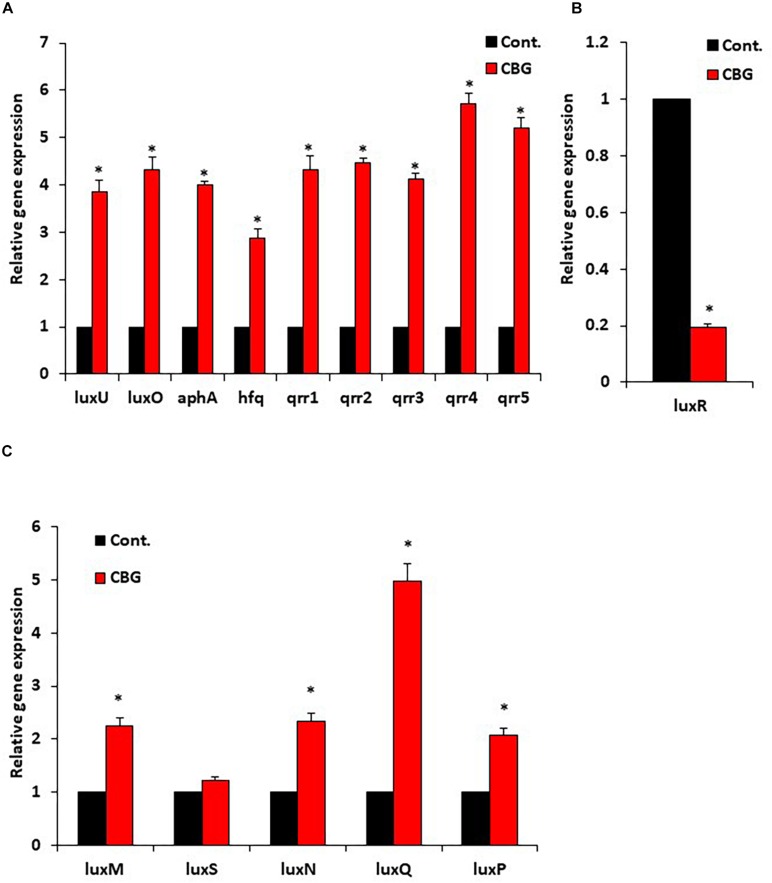
CBG increases the LuxO expression and activity. **(A)** RNA from wild-type *V. harveyi* (BB120) that has been incubated with 2 μg/ml CBG for 10 h were analyzed by real-time PCR for *luxU* and *luxO* gene expression as well as the expression of the LuxO-regulated genes aphA, hfq, and qrr1-5. The relative expression was compared to untreated bacteria incubated for the same time period using 16S rRNA as internal control. **(B)** The relative gene expression of *luxR* in the same samples described in **(A)**. **(C)** The relative gene expression of autoinducer synthases and autoinducer receptors, *N* = 3, *p < 0.05.

Since quorum sensing cascade is tightly regulated, where LuxO antagonizes the master regulator LuxR (Mukherjee and Bassler, 2019), it was querying to explore changes in the expression of quorum sensing-related genes following CBG treatment. To this end, wild-type *V. harveyi* was incubated in the absence or presence of 2 μg/ml CBG for 10 h, and the RNA was extracted for real-time PCR analysis. We observed that both the *luxU* and *luxO* gene expression were strongly induced (x3.5–4.5-fold induction) (Figure 6A). Also, the LuxO-regulated genes *aphA*, *hfq*, and *qrr1-5*, were strongly upregulated (x3-6-fold induction) (Figure 6A), indicating increased LuxO activity in the presence of CBG. The strong increase in the regulatory small RNAs of the *qrr* family, explains the strong repression observed in *luxR* expression (80 ± 2% reduction; Figure 6B; *p* < 0.05). The simultaneous upregulation of AphA further antagonizes LuxR gene and protein expression (Mukherjee and Bassler, 2019). The expression of QS genes coding for autoinducer synthases (*luxM*, *luxS*) and receptors (*luxN*, *luxP*, *luxQ*) are shown in Figure 6C. The *luxQ* gene was highly induced, while *luxM*, *luxN*, and *luxP* were modestly induced. CBG had barely any effect on the *luxS* gene expression (Figure 6C).

### The Use of Quorum Sensing Mutants of *V. harveyi* to Delineate the CBG Action Mechanism

**FIGURE 7:**
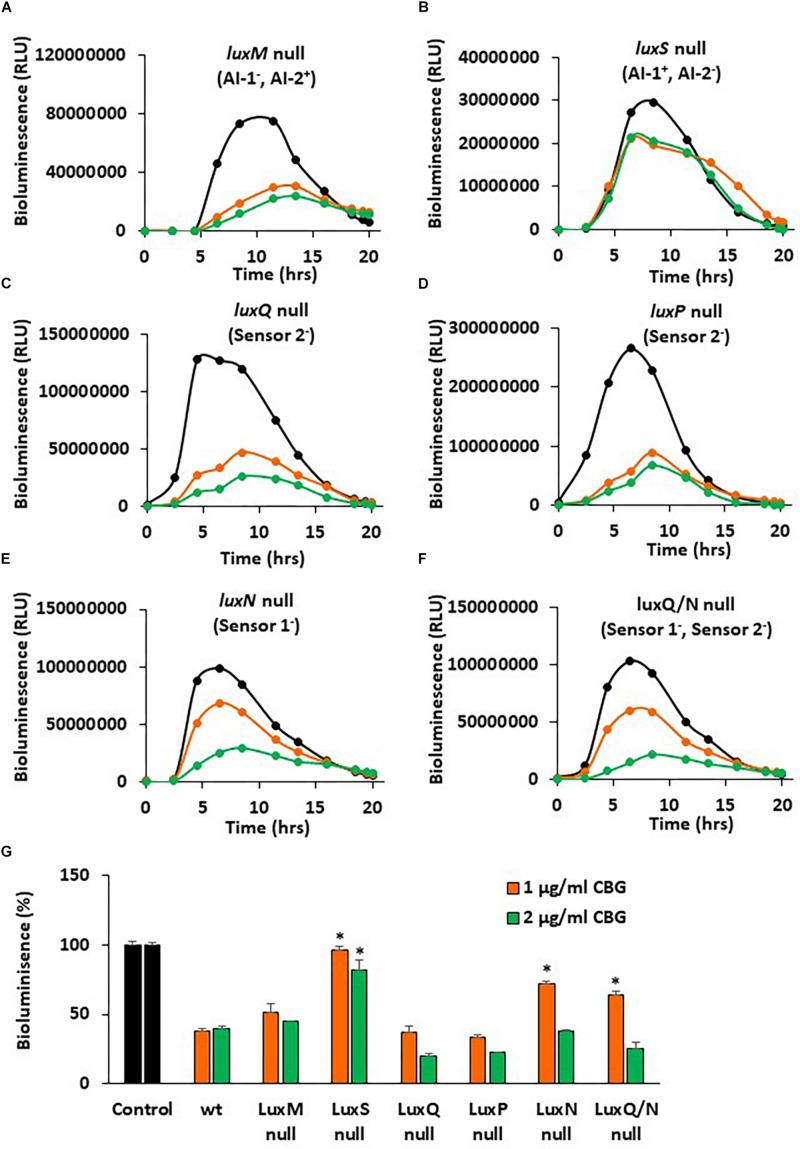
*luxS* null and *luxN* null mutants showed diminished response to CBG. **(A–F)** The bioluminescence of the indicated mutant strains grown in the absence or presence of CBG. The black lines represent control samples of no CBG, the orange lines bacteria treated with 1 μg/ml CBG, and the green lines bacteria treated with 2 μg/ml CBG. Each line is the average of three samples. **(G)** The relative bioluminescence as determined by the area under the curve (AUC) of the graphs presented in **(A–F)**, *N* = 3, *p < 0.05 in comparison to CBG-treated wild-type bacteria.

In a further search for understanding the anti-quorum sensing activity of CBG, we used different mutants of *V. harveyi* deficient in autoinducer production and/or autoinducer receptors. The bioluminescence vs. planktonic growth was studied for each strain in the absence or presence of 1 or 2 μg/ml CBG (Figure 7). The *luxM* null strain lacking AI-1 showed a slightly reduced response to CBG in comparison to wild-type (Figure 7A vs. Figure 2B), whereas the *luxS* null strain lacking AI-2 showed almost no response to CBG (Figure 7B; *p* < 0.05). These findings suggest that CBG predominantly antagonizes AI-2, which is the major autoinducer during the exponential growth phase of *V. harveyi* (Anetzberger et al., 2012). Interestingly, mutants lacking either *luxP* or *luxQ*, which are the two components making up the AI-2 receptor, showed similar, and even stronger, reduction in bioluminescence in response to CBG in comparison to wild-type (Figures 7C,D), suggesting that CBG acts downstream to this receptor. It should be noted that AI-2 acts only on the LuxP/Q receptor in *V. harveyi* (Pereira et al., 2013), and no bioluminescence induction was observed when AI-2 was exogenously added to the *luxP* or *luxQ* null mutants (data not shown). In contrast to the AI-2 receptor null mutants, the mutant lacking *luxN*, the receptor for AI-1, and the mutant lacking both *luxN* and *luxQ*, showed diminished response to CBG (Figures 7E,F; *p* < 0.05), suggesting that LuxN is a major receptor targeted by CBG.

To get a better understanding of the consequences of the elimination of one or more components of the autoinducers and/or their receptors, the relative bioluminescence of each mutant strain was compared. The *luxM* null lacking AI-1 showed a 40% reduction in bioluminescence in comparison to wild-type, while the *luxS* null lacking AI-2, showed more than 98% reduction in bioluminescence (Supplementary Figure S1), again pointing to a major role of AI-2 vs. AI-1. The *luxS*, *luxN* double mutant that are deficient in both the AI-1 and AI-2 pathways showed almost no bioluminescence at all, as expected (Supplementary Figure S1). The *luxP* and *luxN* null mutants showed earlier and stronger bioluminescence (140% increase) than the wild-type (Supplementary Figure S1), a finding that suggests that even in the presence of autoinducers, these receptors still exert, to a certain extent, an agonistic action on LuxU/LuxO (Mukherjee and Bassler, 2019). LuxQ null, showed an earlier onset of bioluminescence, but similar total bioluminescence than the wild-type (Supplementary Figure S1). Of note, the *luxN, luxQ* double knockout mutant showed lower bioluminescence than the *luxN* single null mutant (Supplementary Figure S1), indicating that the two autoinducer receptors act in a mutual regulatory interplay. The latter may explain how can LuxN indirectly affect AI-2 signaling and, in thus, demonstrate how the action of CBG can depend on both AI-2 and LuxN.

## Discussion

Since bacterial biofilm formation is a major virulence factor that impedes the control of pathogenic bacteria and the treatment of pertinent bacterial infections, much research has been conducted to find alternatives to current antibiotic therapy (Asfour, 2018). The quorum sensing system in *V. harveyi* has been extensively studied and quite well defined (Mukherjee and Bassler, 2019), making it a good model system for studying the anti-QS properties of the *Cannabis* compound CBG. The QS system regulates gene expression, including virulence determinants, in response to bacterial cell population density by means of signaling molecules called autoinducers (AIs) (Jiang et al., 2018). Medicinal plants have received recognition as a new source for effective QS inhibitory substances. Several phytochemicals and plant by-products have been acknowledged as QS quenching agents in *V. harveyi* including curcumin, flavonoids and components of cranberry (Feldman et al., 2009; Vikram et al., 2010; Packiavathy et al., 2013; Asfour, 2018).

In the present study, we investigated the effect of CBG on QS-regulated bioluminescence, biofilm formation and motility of *V. harveyi*. We demonstrated that CBG shows strong anti-quorum sensing and anti-biofilm activities on *V. harveyi* at concentrations that do not affect their viability. In addition, CBG caused strong reduction in their motility. So far, the activities of CBG have mainly been studied in eukaryotes where it has been found to be neuroprotective, acting as an anti-oxidant (Giacoppo et al., 2017) and exert anti-inflammatory activities (Eisohly et al., 1982). CBG has previously been found to display anti-bacterial properties toward clinical isolates of methicillin-resistant *Staphylococcus aureus* (Appendino et al., 2008). However, its effect on the bacterial QS pathway has remained unknown.

The QS system in *V. harveyi* activates bioluminescence, and therefore this parameter served to monitor the QS status of the bacteria (Ng and Bassler, 2009; Soni et al., 2015). We conducted kinetic studies that simultaneously monitored bioluminescence and optical densities, allowing for study of the anti-QS effects of CBG. We observed that CBG already at 1 μg/ml exhibited more than 60% inhibition of the bioluminescence of *V. harveyi*. Increasing the concentrations to 20 μg/ml did not significantly augment the anti-quorum sensing effect, suggesting that a plateau effect is reached with 1 μg/ml CBG. The growth of wild-type *V. harveyi* was unaffected by all tested concentrations of CBG, indicating that the effect is specifically directed against the quorum sensing system and not against bacterial viability in the terms of planktonic growth.

Biofilm formation is a major virulence factor that provides the microorganisms with a survival advantage. Therefore, it was important to study the anti-biofilm activity of CBG. We observed that CBG indeed at its sub-MIC concentrations reduced the amount of bacteria in the biofilms and altered the biofilm structure. This indicates a specific anti-biofilm effect of CBG which is unrelated to its anti-bacterial activity.

Quorum sensing has been shown to enhance the flagellar-dependent motility of *V. harveyi* (Yang and Defoirdt, 2015), and the enhanced motility plays a key role in biofilm formation (O’Toole, 2011). Therefore, the effect of CBG on the motility of wild-type *V. harveyi* was investigated. We indeed observed that the motility of *V. harveyi* decreased with increasing concentrations of CBG in a dose-dependent manner. This finding further supports the anti-QS activity of CBG. Next, we were interested in the molecular mechanisms involved in the anti-QS activity of CBG. We first studied whether CBG could antagonize the quorum sensing signals delivered by the autoinducers AI-1 and AI-2. In the first series of experiments, the autoinducers were added exogenously to autoinducer-deficient strains in the absence or presence of CBG. The data obtained from these studies, clearly indicate that CBG can antagonize the QS signals delivered by both AI-1 and AI-2. When using autoinducer mutant strains, we observed that AI-2 had a much stronger impact on bioluminescence than AI-1 under physiological conditions, an observation that accords with previous studies showing that AI-2 is the major autoinducer during the early exponential phase of *V. harveyi* growth, while AI-1 and CAI-1 only appears at later stages (Anetzberger et al., 2012). The *luxS* null (AI-2^–^) mutant showed 98% less basal bioluminescence in comparison to wild-type, although it still emits bioluminescence. The *luxM* null (AI-1^–^) mutant showed a 40% reduction in basal bioluminescence in comparison to wild-type. Interestingly, CBG did not reduce the bioluminescence of the *luxS* null mutant, while still had a significant effect on the *luxM* null mutant. This means that the major anti-quorum sensing effect of CBG acts on interfering with the AI-2-dependent signals.

**FIGURE 8:**
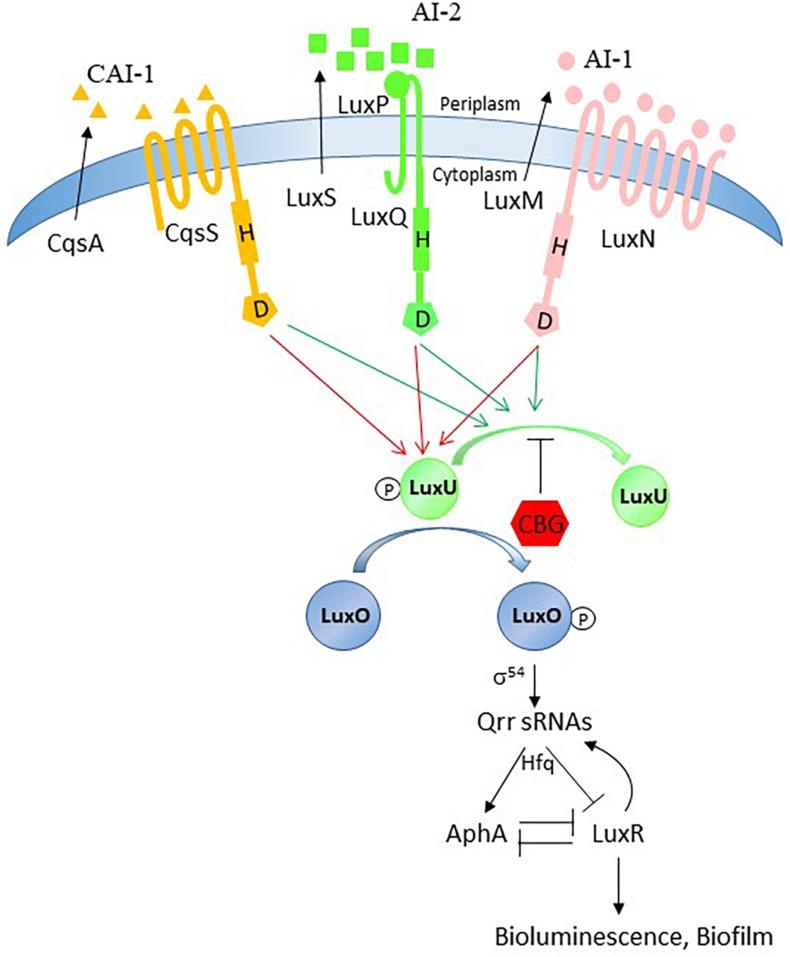
Possible action mechanism of CBG. The quorum sensing system is tightly regulated and has many feedback loops. Our data provide evidence that the anti-quorum sensing activity of CBG is caused by antagonizing the signals delivered by AI-1 and AI-2. Based on our observation that LuxN is required for the anti-QS activity of CBG and CBG augments the LuxO activity, we propose that CBG prevents the dephosphorylation of LuxU.

A surprising result was the observation that the *luxP* and *luxQ* null mutants deficient in the AI-2 receptor still responded well to the anti-QS activity of CBG. These mutants differed from the *luxS* mutant in still showing high bioluminescence, which might be due to the lack of agonism of the AI-2 receptor on LuxU/LuxO. The *luxP* and *luxQ* mutants rely mainly on the AI-1/LuxN for the induction of bioluminescence suggesting that it is this pathway that is targeted by CBG. We observed that the *luxN* null mutant responded less well to CBG than wild-type, providing further support that LuxN is a target of CBG. It is worth noting that the LuxN has nine transmembrane regions (Swem et al., 2008) and CBG is a hydrophobic molecule (Figure 1). It could be presumed that this structure of LuxN may facilitate the transport of the CBG molecules into the bacteria. Another possibility is that CBG increases the agonistic effect of LuxN on LuxU, by either increasing the LuxN kinase activity, or preventing the LuxN-mediated dephosphorylation of LuxU. In both cases, the response regulator LuxO will be kept active and antagonizes the master regulator LuxR involved in biofilm formation and bioluminescence. Indeed, gene expression studies showed that CBG induced the gene expression of *luxU* and *luxO* as well as the LuxO-regulated genes aphA, hfq and the small regulatory RNAs qrr1-5, demonstrating that CBG does increase the LuxO activity in *V. harveyi*. Concomitant with the increased LuxO activity, the *LuxR* gene expression was strongly reduced by CBG, which explains the anti-quorum and anti-biofilm activity of CBG (Figure 8).

In summary, the present study demonstrates the antagonistic effects of the *Cannabis* component CBG on QS-mediated processes in *V. harveyi*, including bioluminescence, biofilm formation and motility, at concentrations that do not affect planktonic growth. CBG antagonizes both the AI-1 and AI-2 pathways, through acting, among others, on LuxN with resulting increase in LuxO activity. The interference of CBG with the bacterial signal-transduction system provides a novel innovative way to combat bacterial biofilm formation.

## Data Availability Statement

The datasets generated for this study are available on request to the corresponding author.

## Author Contributions

MA, RG, RS, BZ, MF, and DS conceived the idea. MA designed and performed the experiments and analyzed the data. MA wrote the paper with RS and DS.

## Conflict of Interest

The authors declare that the research was conducted in the absence of any commercial or financial relationships that could be construed as a potential conflict of interest.

## Abbreviations

AB: Autoinducer bioassay
AI: Autoinducer
CBG: Cannabigerol
DPD: (S)-4,5-Dihydroxy-2,3-pentandione
LCD: low cell density
HCD: high cell density
QS: Quorum sensing.
