# Supplementary figures and images for: Cannabigerol Prevents Quorum Sensing and Biofilm Formation of Vibrio harveyi
Source: Front Microbiol. 2020 May 7;11:858. doi: 10.3389/fmicb.2020.00858 (PMC7221000; doi:10.3389/fmicb.2020.00858)

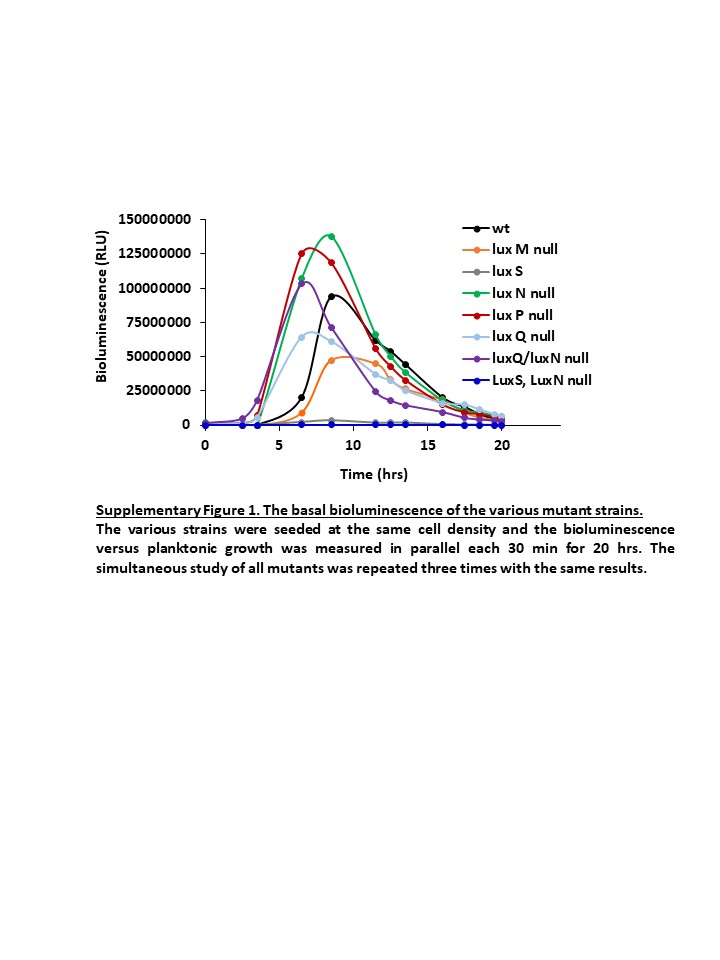

Supplement: Supplementary file 1 [file Image_1.JPEG]
